# Physiological, Histological, and Molecular Responses of White Trevally (*Pseudocaranx dentex*) to Acute Hypoxia

**DOI:** 10.3390/ani16193137

**Published:** 2026-10-07

**Authors:** Xiatian Chen, Nan Zhang, Xiaoming Zhang, Jialing Luo, Fenglin Wang, Yudong Jia

**Affiliations:** Yellow Sea Fisheries Research Institute, Chinese Academy of Fishery Sciences, Qingdao 266071, China; chenxiatian1216@163.com (X.C.); 18526164783@163.com (N.Z.); 18766973648@163.com (X.Z.); luojialing2023@163.com (J.L.); wangfl@ysfri.ac.cn (F.W.)

**Keywords:** *Pseudocaranx dentex*, hypoxia tolerance, oxidative stress, apoptosis, histopathology

## Abstract

Dissolved oxygen (DO) is a key environmental factor affecting the survival and development of farmed fish. However, the hypoxia responses of white trevally (*Pseudocaranx dentex*) remain poorly understood. In this study, we determined the DO tolerance thresholds and integrated physiological, histological, and molecular responses of white trevally to hypoxic stress. Smaller fish exhibited higher values for critical oxygen tension (3.50 ± 0.18 mg/L) and loss of equilibrium (LOE, 0.92 ± 0.14 mg/L), whereas larger fish showed lower values. Hypoxia significantly elevated serum cortisol, glucose, alanine aminotransferase, and aspartate aminotransferase levels, which peaked at LOE. Under hypoxia, histological analysis revealed lamellar clubbing, hypertrophy, and hyperplasia in gills, and vacuolization and nuclear migration in liver sections. In the liver, superoxide dismutase and catalase activities and malondialdehyde content increased, while glutathione peroxidase activity decreased. Upregulation was observed for stress-related genes (*HSP70*, *HSP90*, mineralocorticoid receptor, and glucocorticoid receptor) and pro-apoptotic genes (*p53*, *BAX*, and *Caspase-3*), while *Bcl-2* was downregulated. Principal component analysis indicated that hypoxia triggered stress responses, tissue damage and apoptotic signaling; smaller fish were the most vulnerable, emphasizing the need for size-specific management in aquaculture. Collectively, these findings provide baseline data that may support the healthy development of traditional white trevally aquaculture.

## Simple Summary

White trevally is a marine fish species with high commercial value in aquaculture; however, its response to low oxygen levels remains unclear. In this study, we assessed the hypoxia tolerance of white trevally and characterized its hematological parameters, histological architecture, and gene expression profiles under acute hypoxic stress. Our results revealed that smaller individuals exhibited the highest critical oxygen tension and the earliest onset of loss of equilibrium. Plasma levels of stress hormones and hepatic enzymes increased markedly as dissolved oxygen declined. Gill filaments exhibited lamellar thickening and fusion, whereas hepatocytes displayed cytoplasmic vacuolation and nuclear displacement. Hepatic superoxide dismutase and catalase activities increased, as did malondialdehyde levels, whereas glutathione peroxidase activity decreased. Transcripts related to stress responses and apoptosis were upregulated, whereas an anti-apoptotic gene was downregulated. These findings provide baseline data to guide size-specific oxygen management in white trevally aquaculture, thereby contributing to improved welfare and more sustainable production practices.

## 1. Introduction

Dissolved oxygen (DO) is one of the most critical environmental factors affecting aquatic organisms and profoundly influences their growth, development, and survival [1,2,3]. In intensive aquaculture systems, high stocking densities, elevated water temperatures, and excessive nutrient loading frequently lead to hypoxic conditions. Hypoxia exerts pleiotropic effects on fish, including impaired feed intake and growth performance [4,5], endocrine disruption and elevated stress hormone levels [6], oxidative stress and tissue damage [7,8], as well as altered swimming behavior and increased disease susceptibility [9,10]. Although physiological and behavioral responses to hypoxia have been documented in several teleost species, knowledge of species-specific tolerance thresholds and integrated adaptive mechanisms under varying biological conditions remains limited. Therefore, hypoxia tolerance and its underlying regulatory mechanisms in aquaculture-relevant fishes remain important research areas.

The white trevally is a pelagic marine fish of the family Carangidae that inhabits subtropical and temperate waters of the Western Pacific and is distributed naturally along the coasts of China, Japan, Korea, and Australia [11]. Because of its high muscle protein content, balanced profiles of essential and umami amino acids, and considerable abundance of unsaturated fatty acids, white trevally has long been considered as a premium ingredient for high-grade sushi and sashimi in Japan, supporting the long-standing tradition of both capture fisheries and aquaculture [12,13,14]. With economic development and growing consumer acceptance, market demand for this species has risen substantially in recent years, driving the expansion of farming areas. In particular, breakthroughs in reproductive technologies, including broodstock maturation control, artificial propagation, and larval rearing, have been achieved in China [14,15,16,17].

Previous research on white trevally has mainly addressed sex identification, disease control, and skeletal muscle physiology [18,19,20]. Given its well-separated fast-twitch and slow-twitch muscles, white trevally serves as an excellent model for sustained-swimming teleosts. Wang et al. demonstrated that slow-twitch muscle predominantly relies on aerobic oxidative metabolism, whereas fast-twitch muscle preferentially depends on anaerobic glycolysis and the ATP-PCr phosphagen system for energy supply. These metabolic differences are accompanied by muscle-type-specific expression of myofibrillar protein isoforms, which determine distinct contractile properties [18]. RNA-seq-based transcriptome profiling has generated gene expression atlases for both muscle types, revealing that differentially expressed genes (DEGs) are enriched mainly in pathways related to energy metabolism, sarcomere structure, and cellular organization [14,17]. Genome-wide DNA methylation profiling further showed that differential promoter methylation influences oxygen delivery and contraction kinetics by modulating the expression of myoglobin, troponin, and myosin regulatory light chains. Moreover, hypomethylation of ladybird homeobox protein homolog 1 and erythropoietin, together with the regulation of semaphorin/plexin genes, was found to increase satellite cell abundance and neuromuscular junction remodeling, indicating enhanced regenerative potential in slow-twitch muscle [21]. A comparative analysis of histidine-related compounds (HRCs) across species with different swimming strategies revealed that in white trevally, HRCs predominantly accumulate in fast-twitch muscle and are strongly positively correlated with anaerobic capacity and intramuscular buffering capacity. The transporters *Slc15a4* and *Slc7a2* were identified as potential critical regulators of HRC trafficking [22]. Nevertheless, studies on how environmental factors affect white trevally aquaculture remain limited, particularly regarding the physiological responses of this species to hypoxic stress.

To fill these gaps, we sought to determine the hypoxia tolerance thresholds and associated physiological response patterns of white trevally. Accordingly, fish were exposed to progressively decreasing dissolved oxygen levels under continuous monitoring, and integrative assessments were conducted on swimming behavior, blood biochemistry, hepatic antioxidant capacity, gill and liver histopathology, and stress-response and apoptosis-related genes. This study offers essential data to support the healthy development of white trevally and provides a useful reference for future studies aimed at improving hypoxia tolerance in this species.

## 2. Materials and Methods

### 2.1. Experimental Animals

Juvenile white trevally were obtained from Mingbo Aquatic Products Co., Ltd. (Laizhou, Shandong, China). A total of 270 apparently healthy fish with no visible external abnormalities were selected and assigned to three size classes, small (S; body weight, 50.23 ± 5.67 g; body length, 17.82 ± 0.46 cm), medium (M; body weight, 100.45 ± 8.92 g; body length, 20.86 ± 1.35 cm), and large (L; body weight, 150.78 ± 12.34 g; body length, 23.18 ± 1.29 cm), with 90 fish per size class. Fish within each size class originated from the same batch of fertilized eggs. For each size class, 18 fish were used to determine hypoxia tolerance thresholds, and the remaining 72 fish were subjected to hypoxic stress experiments. Each experimental group was tested in triplicate, with six fish per replicate tank. All fish were acclimated for one week in a recirculating aquaculture system at a stocking density of 25 kg/m^3^, under the following conditions: water temperature 22 ± 1 °C, salinity 29 ± 1‰, dissolved oxygen ≥ 6.5 mg/L, and a 12 h light:12 h dark photoperiod. Fish were hand-fed a commercial compound diet (Haitong, China) twice daily (08:00 and 16:00) until apparent saturation. Fish were fasted for 24 h prior to the experiment.

### 2.2. Determination of Hypoxia Tolerance Thresholds

Critical oxygen tension (Pcrit) and LOE were assessed via a closed-chamber respirometry method. Each fish was placed individually in a respirometry chamber, and the gradual decline in water oxygen partial pressure (PO_2_) was continuously monitored with a dissolved oxygen meter (Aqua TROLL 400, In-Situ Inc., Fort Collins, CO, USA). The oxygen consumption rate (MO_2_) was calculated simultaneously according to the formula [23,24]:

$${MO}_{2}=-\left(\frac{∆DO}{∆t}\right)\times \frac{V}{M}$$

ΔDO/Δt represents the slope of the decrease in dissolved oxygen over the measurement interval, V represents the net water volume of the chamber, and M represents the body mass of the fish (kg). PO_2_ was plotted on the X-axis, and MO_2_ was plotted on the Y-axis via GraphPad Prism (version 10.1.2). Pcrit was determined via segmented linear regression by fitting two intersecting straight lines, and the breakpoint (X0) was recorded as the Pcrit value. The respiratory frequency was measured by counting each opening of the gill cover. The LOE endpoint was defined as the moment when a fish turned laterally or ventrally upward and the corresponding dissolved oxygen concentration was logged.

### 2.3. Hypoxia Stress and Sampling

**Figure 1:**
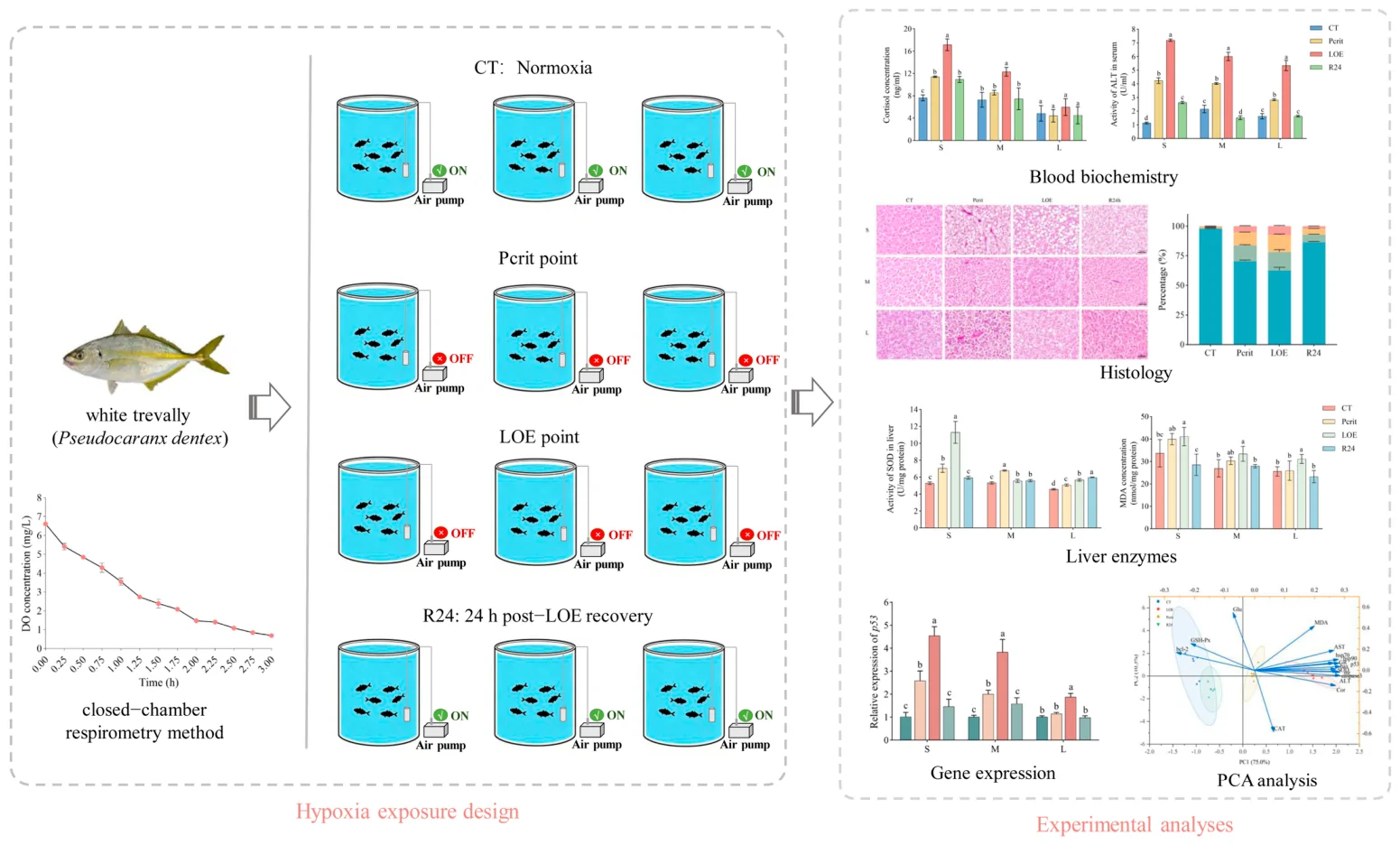
Schematic overview of the hypoxia exposure design and experimental indicator analysis. Different letters above the bars indicate significant differences.

On the basis of the determined Pcrit and LOE values, four treatment groups were established for each size class: a normoxic control group (CT), a Pcrit group, an LOE group, and a 24 h recovery group (R24). For each size class, 72 fish were randomly allocated to these four groups, with each group comprising three replicate tanks and six fish per tank (Figure 1). In the R24 group, the recirculating system was reactivated immediately after LOE was attained, dissolved oxygen was returned to the normoxic level within 30 min, and the fish were allowed to recover for 24 h. Swimming behavior was observed and recorded during the entire exposure. Sampling was performed immediately upon reaching the respective hypoxic endpoint; three fish per replicate were randomly sampled, euthanized with an overdose of MS-222 (200 mg/L, Sigma-Aldrich, St. Louis, MO, USA), and dissected with sterile instruments. Liver and gill tissues were excised, trimmed into appropriately sized pieces, and preserved in 4% paraformaldehyde (Cat# G1101, Servicebio, Beijing, China) and in liquid nitrogen, respectively.

### 2.4. Blood Parameter Assays

Blood was drawn from the caudal vein via a sterile syringe, transferred into tubes and then centrifuged at 3000 rpm for 10 min at 4 °C, after which the serum was collected. The cortisol concentration, glucose concentration, and alanine aminotransferase (ALT) and aspartate aminotransferase (AST) activities were measured via commercial reagent kits (Servicebio, Beijing, China: cortisol, Cat# SEKSM-0071; glucose, Cat# BC2505; ALT, Cat# BC1565; AST, Cat# BC1555) following the manufacturer’s protocols. The absorbance was detected with a microplate reader (SuPerMax 3100, Shanpu, Shanghai, China).

### 2.5. Hepatic Enzyme Activity and Malondialdehyde Content

The activities of superoxide dismutase (SOD), catalase (CAT), and glutathione peroxidase (GSH-Px) and the content of malondialdehyde (MDA) in the liver were determined by corresponding assay kits (Servicebio, Beijing, China: SOD, Cat# BC5165; CAT, Cat# BC0205; GSH-Px, Cat# BC1195; MDA, Cat# BC0025). Briefly, 0.1 g of liver tissue was accurately weighed, homogenized in 1 mL of extraction buffer on ice, and centrifuged at 8000 rpm at 4 °C for 10 min. The supernatant was collected, and the appropriate reagents were added according to the kit instructions. The absorbance was measured at the specified wavelengths with a microplate reader (SuPerMax 3100, Shanpu, Shanghai, China), and the concentrations or enzyme activities were calculated.

### 2.6. Hematoxylin–Eosin Staining

Tissues fixed in 4% paraformaldehyde were dehydrated through a graded ethanol series, cleared in xylene, and infiltrated with paraffin via an automatic tissue processor (Kuohai, Xiaogan, China). The embedded tissue blocks were sectioned at a thickness of 5 μm with a rotary microtome (Leica Microsystems, Wetzlar, Germany). Sections were stained with hematoxylin and eosin (HE). Fish from each replicate were subjected to tissue sectioning. The sections were examined under a light microscope (Leica Microsystems, Wetzlar, Germany), and 20 randomly selected fields per sample were captured and analyzed via ImageJ software (version 1.52a).

### 2.7. RNA Extraction

Total RNA was isolated from approximately 0.1 g of liver tissue that had been snap-frozen in liquid nitrogen immediately after dissection. Extraction was performed via the use of TRIzol reagent (Cat# R401, Vazyme, Nanjing, China) according to the manufacturer’s protocol. Specifically, each sample was homogenized in 1 mL of TRIzol with a tissue homogenizer until fully disrupted. Following homogenization, 200 μL of chloroform was added, and the mixture was vigorously vortexed for 15 s, incubated at 4 °C for 5 min, and centrifuged at 12,000 rpm for 15 min at 4 °C to achieve phase separation. The upper aqueous phase was carefully transferred to a fresh RNase-free tube, and an equal volume of isopropanol was added. The sample was then incubated at 4 °C for 10 min to precipitate the RNA, after which the RNA was pelleted by centrifugation at 12,000 rpm for 10 min at 4 °C. The resulting pellet was washed twice with 75% ethanol, air-dried briefly, and dissolved in nuclease-free water. The purity and concentration of the extracted RNA were assessed via a NanoDrop 2000 spectrophotometer (Thermo Fisher Scientific, Waltham, MA, USA). RNA samples with A_260_/A_280_ ratio between 1.8 and 2.2 and an A_260_/A_230_ ratio ≥ 2.0 were used for the following experiments.

### 2.8. cDNA Synthesis and Quantitative Real-Time PCR (qPCR)

A total of 1 µg of RNA was extracted via the HiScript II Q RT SuperMix for qPCR Kit (Cat# R312, Vazyme, Nanjing, China) to generate cDNA according to the manufacturer’s instructions. qPCR was carried out with the ChamQ Universal SYBR qPCR Master Mix (Cat# Q711, Vazyme, Nanjing, China). Each 20 µL amplification reaction consisted of 1× ChamQ Universal SYBR qPCR master mix, 200 nM each forward and reverse primer, 10 ng of cDNA template, and nuclease-free water to the final volume. The reactions were performed on a real-time quantitative thermal cycler (MA6000, Amolarray, Suzhou, China) under the following thermal profile: initial denaturation at 95 °C for 30 s, followed by 40 cycles of 95 °C for 10 s and 60 °C for 30 s. Amplification specificity was verified by melt curve analysis (95 °C for 15 s, 60 °C for 60 s, and 95 °C for 15 s). All the assays were performed in triplicate. Relative gene expression was calculated via the 2^−ΔΔCt^ method, and *GAPDH* was used as the reference gene to normalize the expression values [14]. The sequences of primers used in this study are listed in Table 1.

### 2.9. Statistical Analysis

All experiments were performed with a minimum of three independent replicates, and the data are presented as the means ± standard deviations (SD). The data were analyzed via IBM SPSS Statistics 27.0 (IBM Corp., Armonk, NY, USA). The Shapiro–Wilk test was used to examine the normality of the data distribution for each group, and Levene’s test was used to assess the homogeneity of variance. Since the data fulfilled both assumptions, one-way analysis of variance was used to evaluate group differences. When a significant overall effect was detected, Tukey’s honestly significant difference test was conducted for post hoc pairwise comparisons. Principal component analysis (PCA) was performed with Origin Pro software (version 2019b). The components with eigenvalues greater than 1 were retained, and their cumulative variance contribution exceeded 70% of the total variance. Statistical significance was set at *p* < 0.05 for all analyses.

## 3. Results

### 3.1. Hypoxic Tolerance Thresholds of White Trevally

**Figure 2:**
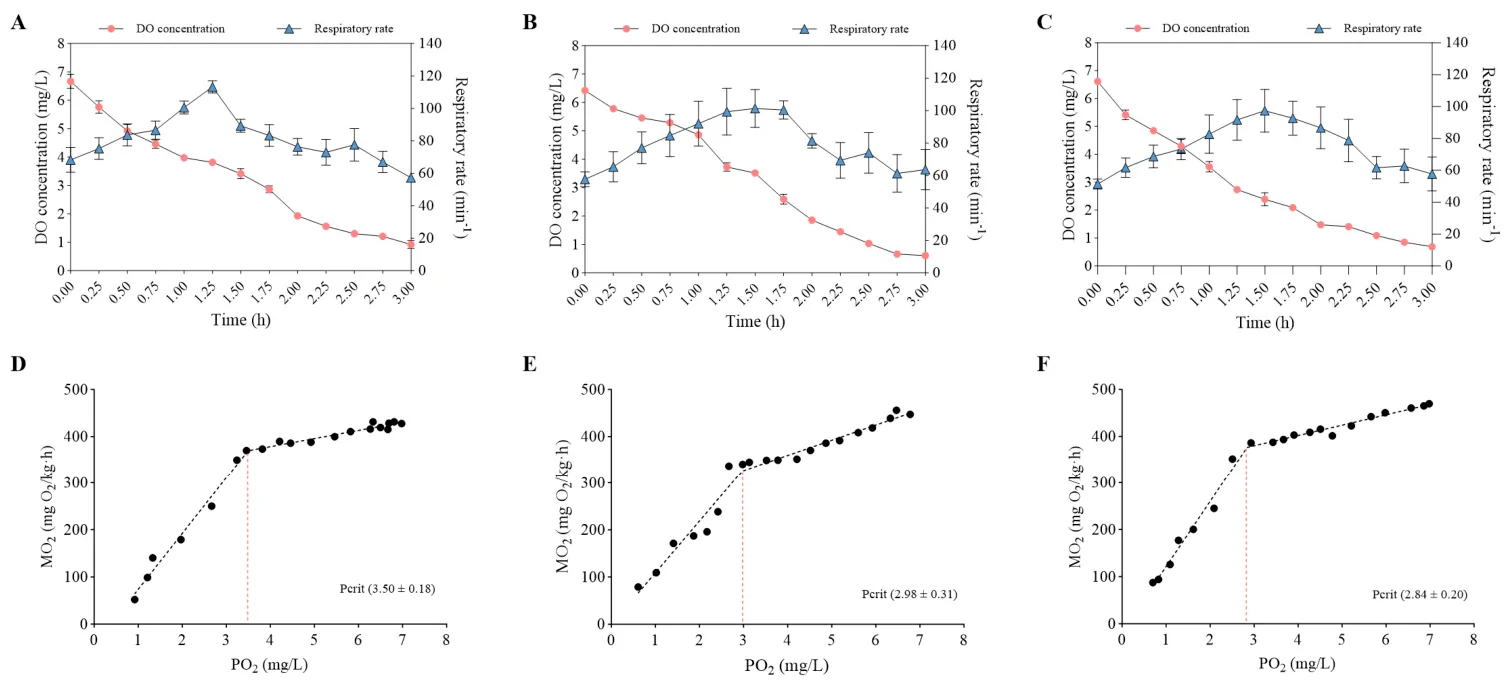
Changes in the DO concentration, respiratory rate, and MO_2_ during the experiment. (**A**–**C**) Time courses of the DO concentration (red line with circle markers, left Y-axis) and respiratory rate (blue line with triangle markers, right Y-axis) in the S (**A**), M (**B**), and L (**C**) groups over 3 h. (**D**–**F**) Relationships between MO_2_ and PO_2_ in the S (**D**), M (**E**), and L (**F**) groups. The black dots represent the calculated data, and the black dotted lines represent the fitted trend lines. The vertical red dashed lines indicate Pcrit, with the corresponding Pcrit values shown below each graph.

During progressive hypoxia, the respiratory rate of white trevally across the three size classes initially increased but subsequently decreased (Figure 2). The S group exhibited the most pronounced initial increase, with the peak ventilation rate occurring at approximately 3.8 mg/L DO, whereas the M and L groups reached peak ventilation at lower DO concentrations. LOE occurred at DO concentrations of 0.92 ± 0.14, 0.61 ± 0.11, and 0.69 ± 0.03 mg/L for the S, M, and L groups, respectively. The Pcrit values were 3.50 ± 0.18, 2.98 ± 0.31, and 2.84 ± 0.20 mg/L for the S, M, and L groups, respectively.

### 3.2. Hematological Responses of White Trevally to Hypoxic Stress

**Figure 3:**
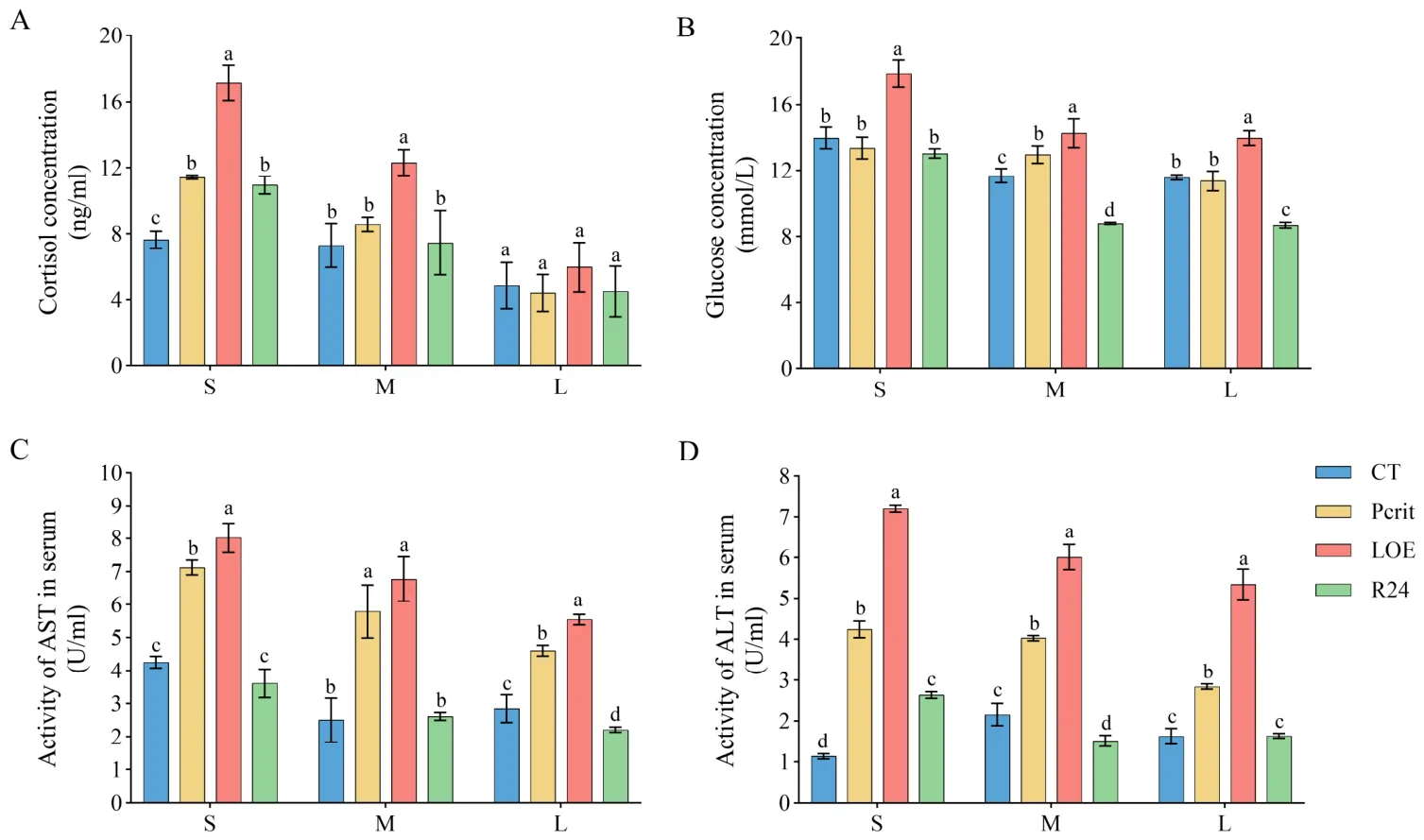
Serum biochemical parameters of white trevally subjected to hypoxic conditions. (**A**) Serum cortisol concentration. (**B**) Serum glucose concentration. (**C**) Serum AST activity. (**D**) Serum ALT activity. Different letters above the bars indicate significant differences. S: small-size group. M: medium-size group. L: large-size group.

To characterize the stress response elicited by hypoxia, serum cortisol levels were measured. As shown in Figure 3A, hypoxic exposure caused a pronounced elevation in circulating cortisol in the S and M groups, with values peaking at the LOE sampling point (approximately 2.24-fold and 1.68-fold above control levels, respectively). This response was most pronounced in the S group. In the L group, cortisol showed an increasing trend, but the difference did not reach statistical significance. Serum glucose displayed a pattern comparable to that of cortisol in all three size classes, with the highest values at LOE. Relative to the controls, glucose at LOE increased by approximately 1.28-, 1.22-, and 1.20-fold in the S, M, and L groups, respectively, and the strongest increase occurred in the S group. Notably, following 24 h of recovery, glucose concentrations in the M and L groups were significantly lower than those in the corresponding CT fish (Figure 3B).

To further assess potential tissue damage, we measured the serum activities of ALT and AST. Hypoxic stress elevated the activities of both enzymes across all size groups, with ALT showing a more marked response and peak activity at the LOE stage (Figure 3C,D). Among the three size classes, the S group exhibited the greatest changes, with ALT activity reaching approximately 7-fold the control level. Apart from the S group, enzyme activities in the other groups returned to control levels after 24 h of recovery.

### 3.3. Gill Histopathological Responses to Hypoxic Stress

**Figure 4:**
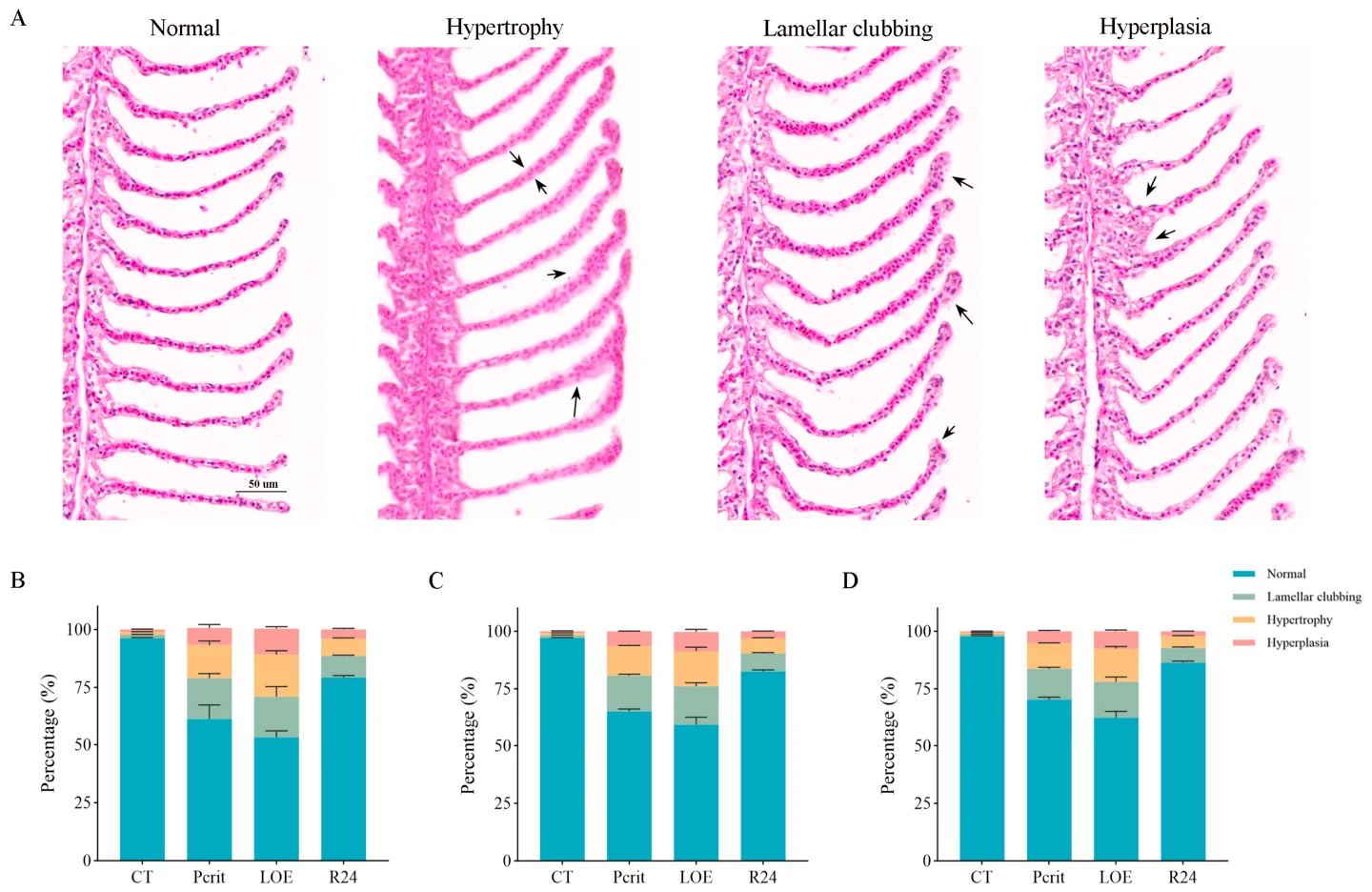
Morphological analysis and quantification of abnormal gill tissue. (**A**) Representative images of normal and abnormal gill morphology. The black arrows indicate abnormal sites. (**B**–**D**) Quantitative analysis of the proportion of abnormal gill tissue area in white trevally of S, M, and L sizes, respectively. Bar = 50 μm.

Histological examination of H&E-stained gill sections revealed that hypoxic stress markedly disrupted normal gill morphology in white trevally, as evidenced by lamellar clubbing, hypertrophy, and hyperplasia (Figure 4A). At Pcrit, the percentages of gill lesions were approximately 39%, 35%, and 30% in the S, M, and L groups, respectively. Under LOE conditions, the incidence of gill lesions increased further, reaching 47%, 41%, and 38% in the S, M, and L groups, respectively. Among the observed lesion types, lamellar clubbing was the most prevalent, followed by hypertrophy and hyperplasia. After 24 h of recovery, gill morphology was substantially restored, with normal tissue proportions reaching 79%, 82%, and 86% in the S, M, and L groups, respectively (Figure 4B–D).

### 3.4. Hepatic Histopathological and Functional Responses to Hypoxic Stress

**Figure 5:**
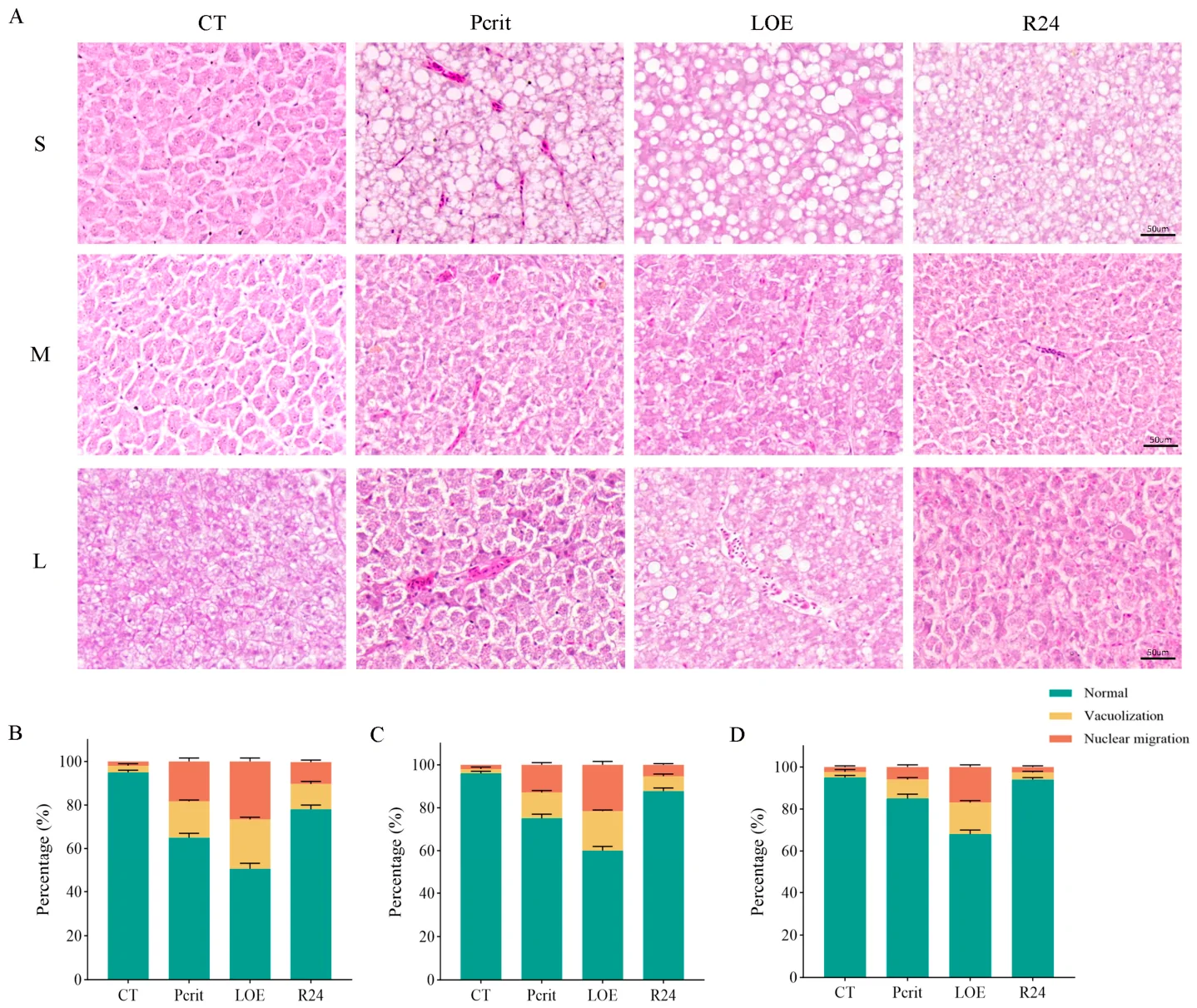
Morphological analysis and quantification of liver tissues. (**A**) Representative liver histology of white trevally of different sizes under different treatment conditions. (**B**–**D**) Quantitative analysis of the proportions of abnormal liver tissue areas in white samples of S, M, and L sizes, respectively. S: small-size group; M: medium-size group; L: large-size group. Bar = 50 μm.

Histological examination of liver sections revealed that hypoxic stress induced hepatocyte vacuolization and nuclear migration in white trevally (Figure 5A). At the Pcrit point, hepatic abnormalities were evident in all three size classes, with the highest proportion observed in the S group (35%) and the lowest in the L group (15%). Under LOE conditions, the proportion of affected hepatocytes increased substantially, reaching approximately 50%, 40%, and 32% in the S, M, and L groups, respectively. After 24 h of recovery, the hepatic lesions were alleviated; however, the S group still presented a relatively high proportion of abnormal hepatocytes (22%) (Figure 5B–D).

**Figure 6:**
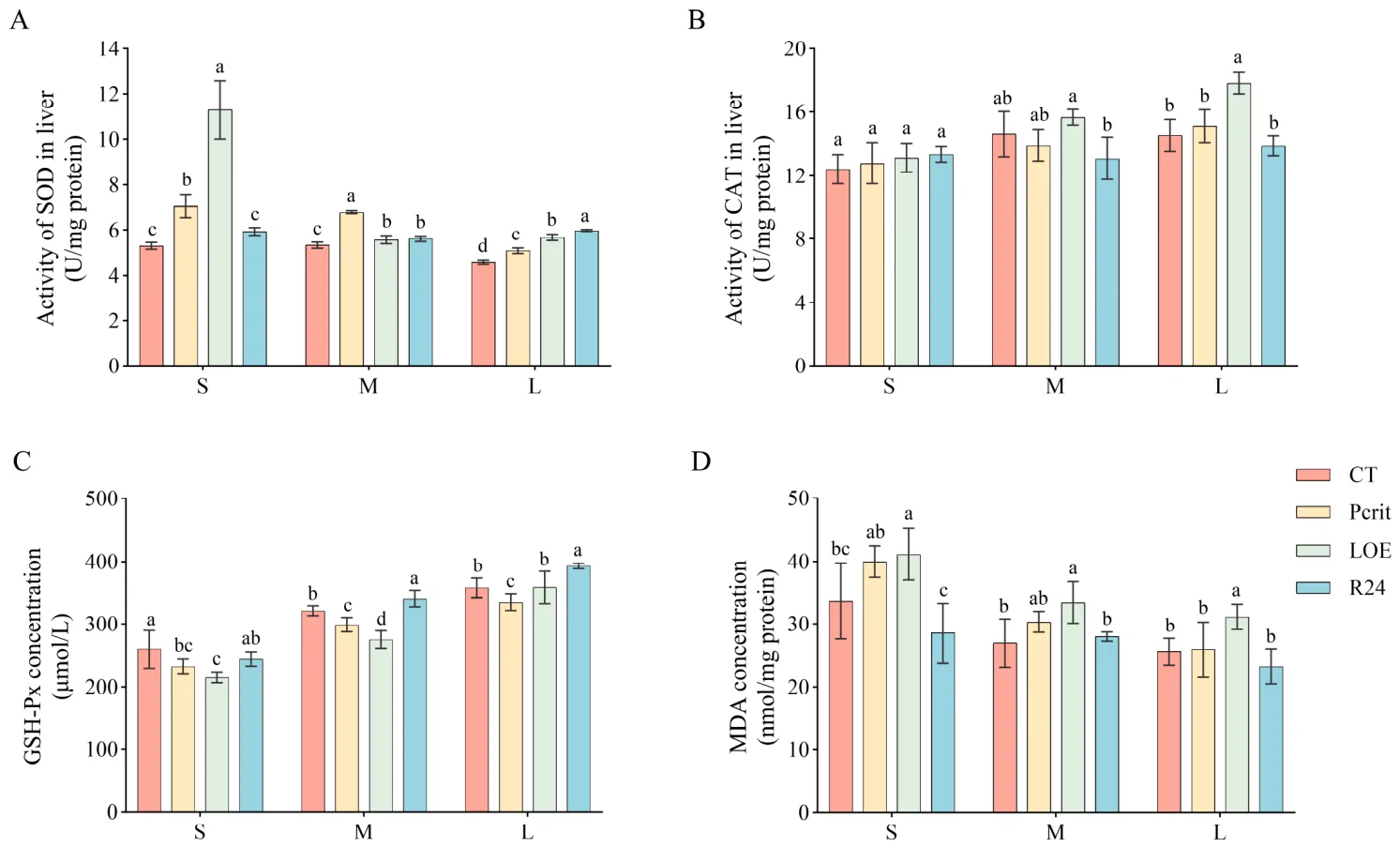
Hepatic antioxidant parameters. (**A**) SOD activity. (**B**) CAT activity. (**C**) GSH-Px content. (**D**) MDA content. S: small-size group; M: medium-size group; L: large-size group. Different letters above the bars indicate significant differences.

To further evaluate hepatic function, the activities of SOD, CAT, and GSH-Px, as well as the MDA content, were determined. SOD activity increased under hypoxic stress, with the most pronounced increase in the S group at LOE (approximately 2-fold greater than that of the control). In the M group, SOD activity at the Pcrit point was greater than that at the LOE point (Figure 6A). CAT activity also tended to increase; however, the changes did not reach statistical significance in the S and M groups, whereas in the L group, the highest CAT activity was observed at LOE (Figure 6B). GSH-Px activity decreased under hypoxia in all three size classes, with the lowest levels recorded in the S group. Following recovery, the GSH-Px activity in the S group did not differ significantly from that in the control group, whereas the activities in the M and L groups were significantly greater than those in the control groups (Figure 6C). The MDA content tended to increase under hypoxia, with the most pronounced change in the S group, reaching approximately 1.2-fold greater than that of the control at LOE. The MDA levels were also significantly elevated at LOE in the M and L groups. After 24 h of recovery, the MDA content did not differ significantly from that of the control in any group (Figure 6D).

### 3.5. Hepatic Gene Expression Responses to Hypoxic Stress

**Figure 7:**
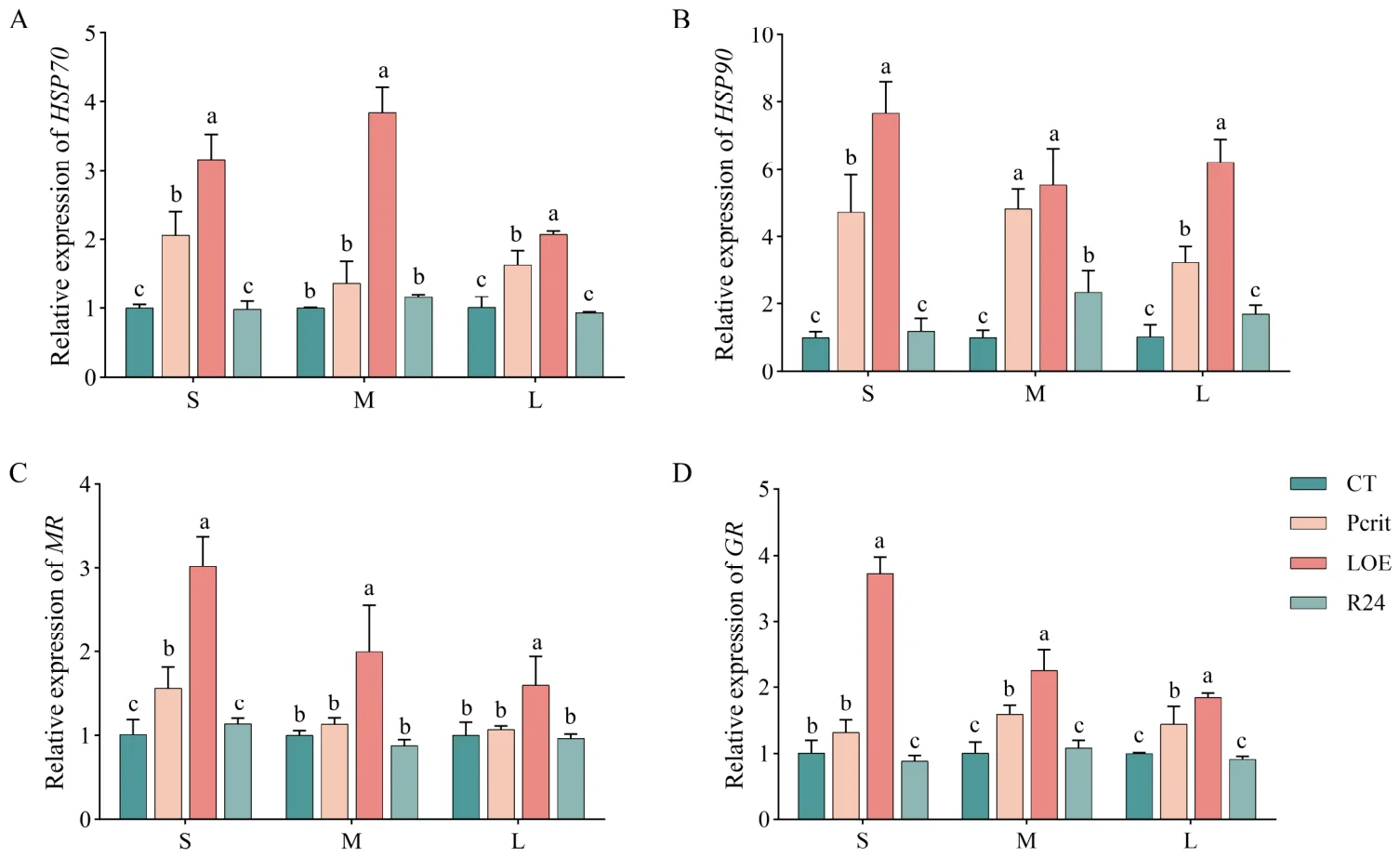
Expression of stress-related genes in the liver. (**A**) *HSP70* mRNA expression. (**B**) *HSP90* mRNA expression. (**C**) *MR* mRNA expression. (**D**) *GR* mRNA expression. S: small-size group; M: medium-size group; L: large-size group. Different letters above the bars indicate significant differences.

To further investigate the molecular processes underlying the observed stress responses, the hepatic expression of stress-related genes, including *HSP*s, *MR*, and *GR*, was quantified. Hypoxic exposure upregulated the expression of both *HSP70* and *HSP90*, with peak levels observed at the LOE point in all size classes (Figure 7A,B). *HSP90* expression was consistently higher than that of *HSP70*; in the S group, *HSP90* and *HSP70* were upregulated approximately 7-fold and 3-fold, respectively, relative to the control. The M and L groups showed similar expression patterns. After 24 h of recovery, the expression of both HSP genes did not differ significantly from that of the control. *MR* expression was also upregulated under hypoxia, with the greatest change observed in the S group (approximately 3-fold at LOE) and the smallest in the L group (Figure 7C). In the M and L groups, *MR* expression did not change significantly at the Pcrit point, and *MR* expression in the recovery groups did not differ significantly from that in the control. *GR* expression followed a pattern similar to that of *MR*, with the largest increase in the S group (approximately 3.6-fold at LOE), followed by the M group (approximately 2.2-fold) and the L group (approximately 1.9-fold) (Figure 7D).

**Figure 8:**
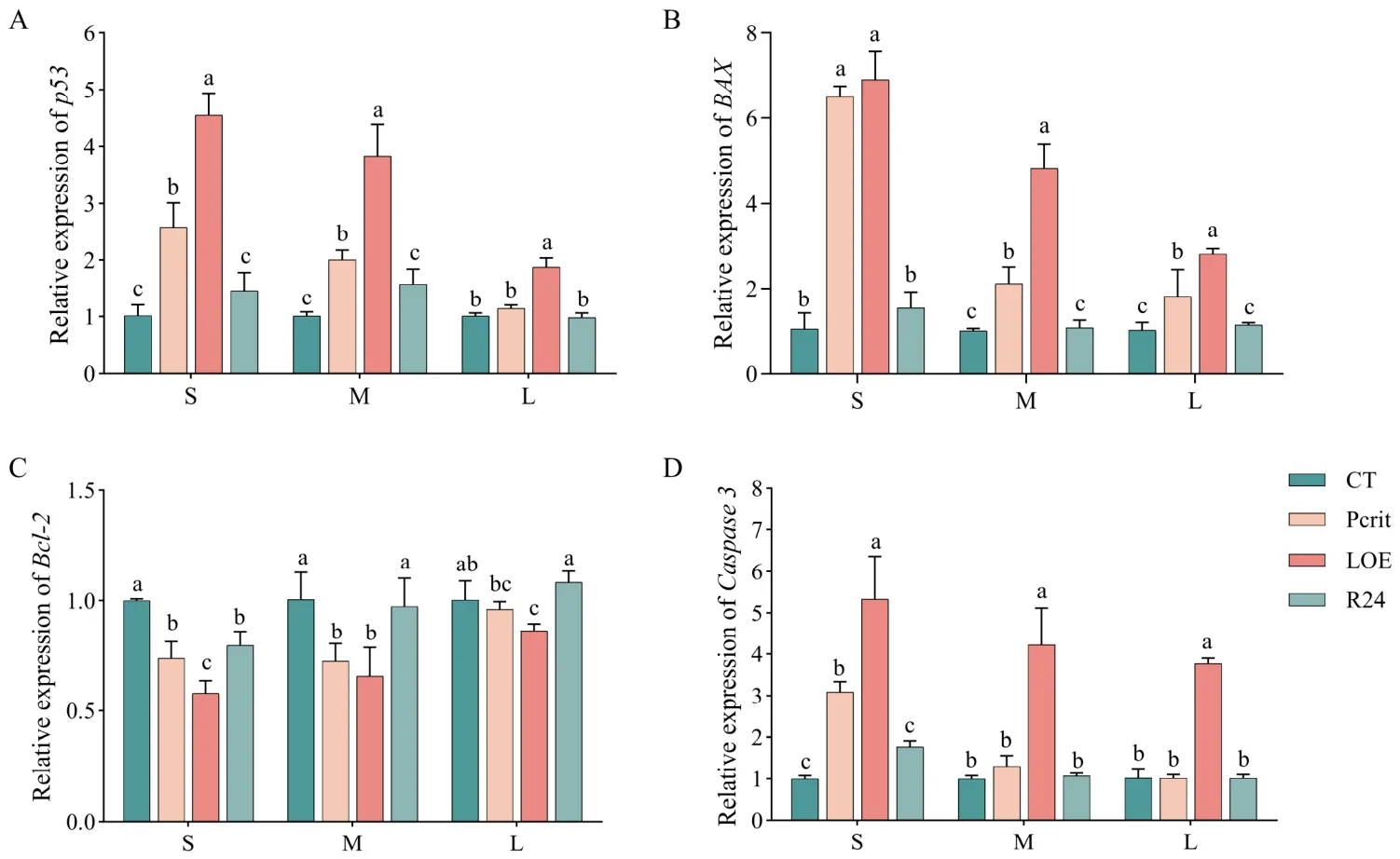
Expression of apoptosis-related genes in the liver. (**A**) *p53* mRNA expression. (**B**) *BAX* mRNA expression. (**C**) *Bcl-2* mRNA expression. (**D**) *Caspase-3* mRNA expression. S: small-size group; M: medium-size group; L: large-size group. Different letters above the bars indicate significant differences.

Apoptosis-related gene expression was also examined in this study. *p53* expression was significantly upregulated under hypoxic stress, with fold changes at LOE of approximately 4.5, 3.7, and 1.9 in the S, M, and L groups, respectively (Figure 8A). *BAX* expression tended to increase, with a sharp rise observed at the Pcrit point in the S group (approximately 6.2-fold greater than that in the control). In the M and L groups, *BAX* expression increased significantly under hypoxia, peaking at LOE, and returned to control levels after recovery (Figure 8B). In contrast, *Bcl-2* expression was downregulated under hypoxia, reaching minimum levels at LOE in all size classes (0.58-, 0.65-, and 0.86-fold relative to CT in the S, M, and L groups, respectively). Notably, in the S group, *Bcl-2* expression remained below control levels after recovery (Figure 8C). *Caspase-3* expression was upregulated under hypoxia, with significant upregulation in the S group; in the M and L groups, no significant alteration was detected at Pcrit, whereas significant upregulation was observed at LOE (Figure 8D).

### 3.6. PCA of Hypoxia-Induced Physiological and Molecular Responses

**Figure 9:**
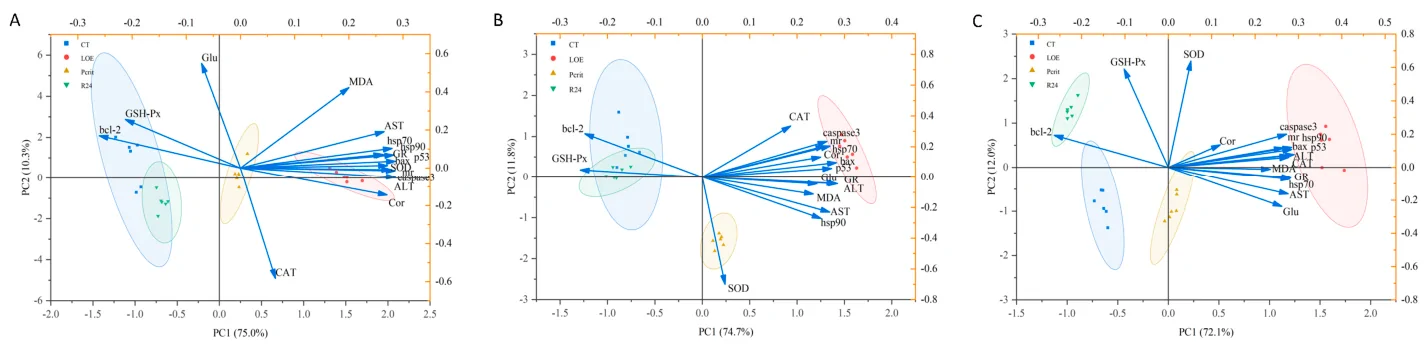
PCA of hypoxia-induced physiological and molecular responses. (**A**–**C**) PCA score plots for the S (**A**), M (**B**), and L (**C**) groups. The X- and Y-axes denote PC1 and PC2, with the percentages of variance explained shown in parentheses. The colored symbols (dots and triangles) represent individual samples from the four treatment groups (CT, Pcrit, LOE and R24), and the colored ellipses indicate the distribution range of each group. The blue arrows indicate loading vectors of physiological and biochemical indicators, reflecting their contributions to the principal components and correlations with sample groups. The full names of the physiological and biochemical indicators are defined in the main text.

As shown in Figure 9, in all the groups, the first principal component (PC1) clearly separated the CT and R24 samples, which clustered on the negative side of PC1, from the Pcrit and LOE samples, which dispersed along the positive side. Positive PC1 loadings were characterized by proapoptotic factors (*p53*, *Caspase-3*, and *BAX*), heat shock proteins (*HSP70* and *HSP90*), liver injury markers (AST and ALT), cortisol, and MDA. Conversely, the CT and R24 groups were concentrated in the negative region of PC1 and tended to distribute along the antiapoptotic protein *Bcl-2* and the antioxidant enzyme GSH-Px. In the S class, the Pcrit group occupied an intermediate position between the CT and LOE groups along PC1 and showed no marked deviation on PC2, whereas the LOE group was additionally positively associated with SOD, CAT, and *GR* vectors. In the M class, the Pcrit group shifted independently in the negative direction of PC2, and loading analysis indicated that this shift was specifically associated with elevated SOD expression. In the L class, although the R24 group generally returned to the CT cluster, the distribution patterns of R24 samples along the *Bcl-2*, GSH-Px, and SOD vectors were deviated slightly from those of the CT group, suggesting that the antiapoptotic and antioxidant baselines were not fully restored after recovery.

## 4. Discussion

Hypoxia is a critical environmental stressor in aquaculture, and fish of different body sizes often exhibit distinct hypoxia tolerances [25,26]. In the present study, the 50 g group exhibited significantly higher Pcrit (3.50 ± 0.18 mg/L) and LOE (0.92 ± 0.14 mg/L) values than the 100 g (Pcrit: 2.98 ± 0.31 mg/L; LOE: 0.61 ± 0.11 mg/L) and 150 g (Pcrit: 2.84 ± 0.20 mg/L; LOE: 0.69 ± 0.03 mg/L) groups, indicating that smaller white trevally are more sensitive to hypoxia. These differences may be attributed to better-developed gill filaments, higher hemoglobin content, and greater oxygen-carrying capacity in larger fish, whereas smaller fish have higher mass-specific metabolic rates and oxygen consumption, making them more susceptible to oxygen limitation and energy imbalance under hypoxia [6,27,28]. These results indicate that the hypoxia tolerance of white trevally is strongly size-dependent, with smaller individuals being more vulnerable.

Cortisol and glucose are key physiological indicators of stress and energy metabolism in fish. Hypoxia activates the hypothalamic–pituitary–interrenal axis, promoting cortisol secretion and thereby stimulating glycogenolysis and gluconeogenesis, leading to elevated plasma glucose [29,30,31]. Our results showed that hypoxia significantly increased serum glucose levels in all size groups, whereas the increase in serum cortisol was not statistically significant in the L group. Notably, the S group exhibited the largest response, and after 24 h of recovery, glucose levels in the M group decreased significantly below control values, suggesting a possible overcompensation metabolic disturbance. These findings are consistent with previous reports in juvenile stellate sturgeon (*Acipenser stellatus*) [32]. The stronger stress response and incomplete recovery observed in small fish may be attributable to their less developed stress regulatory systems. Overall, hypoxia elicits marked stress responses and energy mobilization in white trevally, with small individuals exhibiting greater sensitivity and slower recovery, underscoring the need for size-specific management.

To further assess whether hypoxia induced tissue damage, serum ALT and AST activities were measured. Hypoxic stress significantly elevated both serum ALT and AST activities. These findings are consistent with previous studies on crucian carp (*Carassius auratus*) [33], black rockfish (*Sebastes schlegelii*) [34], and fat greenling (*Hexagrammos otakii*) [24], further suggesting that hypoxia can induce tissue damage and compromise fish survival. The gills and liver are vital organs for maintaining normal physiological function in fish. H&E staining revealed marked pathological alterations in both the gill and liver tissues of white trevally under hypoxic stress, with the most severe lesions in the S group, followed by those in the M group, and the mildest lesions in the L group. In parallel, hepatic function parameters, including SOD, CAT, and GSH-Px activities, as well as MDA content, were significantly altered, indicating disrupted redox homeostasis. Overall, the histopathological and biochemical results demonstrated that the S group sustained the most severe damage, and certain stress indicators remained elevated even after 24 h of recovery. These results suggest that small white trevally possess a relatively weak hepatic antioxidant capacity and are relatively susceptible to oxidative damage, highlighting the importance of ensuring an adequate oxygen supply in the culture management of small fish.

Environmental stress induces not only physiological and biochemical alterations but also molecular stress responses [35,36,37]. The preceding results showed that hypoxic stress significantly induced oxidative stress in tissues. In this study, *HSP90* and *HSP70* were significantly upregulated under hypoxia, with *HSP90* showing greater induction than *HSP70*. *GR* is a nuclear receptor that modulates gene expression through transcriptional activation of metabolic genes and transcriptional repression of pro-inflammatory pathways, representing a central hub in the stress response [38]. qPCR analysis revealed that both *GR* and *MR* were significantly upregulated under hypoxia, indicating excessive activation of corticosteroid receptors, which is consistent with the physiological and biochemical alterations described above. Oxidative stress is frequently accompanied by excessive activation of apoptosis; therefore, the expression of apoptosis-related genes was further examined. The results revealed that hypoxia significantly upregulated the pro-apoptotic genes *BAX*, *p53*, and *Caspase-3*, with expression levels increasing with increasing stress severity, whereas the anti-apoptotic gene *Bcl-2* was downregulated. After 24 h of recovery, the expression of these genes largely returned to control levels. These molecular alterations are consistent with previous reports in large yellow croaker (*Larimichthys crocea*), Nile tilapia (*Oreochromis niloticus*) and grass carp (*Ctenopharyngodon idella*) [39,40,41].

PCA provides an integrated perspective on the physiological and molecular responses to hypoxia. The results revealed that severe hypoxia broadly activated apoptotic cascades and stress signaling pathways, resulting in marked hepatic metabolic impairment, whereas the recovery group exhibited increased anti-apoptotic and antioxidant effects, facilitating the reestablishment of physiological homeostasis, with the levels of these genes approaching control levels. Moreover, hypoxia-induced damage was more pronounced in small fish.

## 5. Conclusions

In conclusion, this study demonstrated that hypoxia tolerance in white trevally is size-dependent, with small individuals being more vulnerable to low dissolved oxygen than medium and large individuals. Hypoxic exposure elicited a coordinated stress response characterized by elevated serum cortisol and glucose levels, hepatic oxidative stress, and gill and liver tissue damage. At the molecular level, hypoxia upregulated heat shock proteins, corticosteroid receptors, and pro-apoptotic genes (*p53*, *BAX*, and *Caspase-3*) while downregulating the gene *Bcl-2*, indicating the activation of apoptotic signaling. PCA further revealed that small fish exhibited stronger stress and apoptotic signatures under hypoxic conditions. These findings provide comprehensive insights into the hypoxia tolerance responses of white trevally and highlight the importance of size-specific management strategies for mitigating hypoxia-induced losses in aquaculture. However, these conclusions are derived exclusively from the present experimental dataset, and future investigations are necessary to validate the conclusions drawn from this study.

## Figures and Tables

**Table 1:** Information on the primers used for qPCR in this study.

**Gene Name**	**Primer**	**Sequences**	**Tm (°C)**	**Product Length (bp)**	**Accession Number**
*MR*	Forward	AGAATAACAGCGTGCCCACA	59.96	102	XM_044347227.1
	Reverse	GCCGCGTGAAAGACATTGTT	60.04		
*GR*	Forward	CTCCCAGTAACGACCATCGG	59.90	183	XM_013271703.3
	Reverse	AGTCCCGCTCTTTCCTTGTG	59.96		
*HSP70*	Forward	GACCTCTTCAGGGGAACACTA	58.46	101	NM_001113589.1
	Reverse	GTTGATCCCCCAACCAGCAC	61.25		
*HSP90*	Forward	AAGTGTCTCGAGCTCTTCGC	60.11	120	XM_051409352.1
	Reverse	CTTCTTGCGGTTCTGGGAGT	59.96		
*p53*	Forward	CCCGGAGGTGTCCACTAAAG	59.75	107	XM_044340808.1
	Reverse	CGGACTTCTGAAAACGCAGC	60.11		
*BAX*	Forward	GCGGTGGATGCTGACAGAAA	60.96	126	XM_073943491.1
	Reverse	AGAAGTGCAGCTCCCAAGTC	59.96		
*Bcl-2*	Forward	CACAACCTGTTCCTGACCGT	60.18	113	XM_044065198.1
	Reverse	CGGTTAACGCTCTCCACACA	60.32		
*Caspase-3*	Forward	ATGACCAGACAGTCGAGCAG	59.97	131	XM_051385103.1
	Reverse	GCCGTCCGTACCAAAGAACA	60.60		
*GAPDH*	Forward	CTACCACGGTGAGGTCAAGG	59.75	178	XM_044360213.1
	Reverse	GCCCTTCAGATGAGCAGAGG	60.18		

*MR*: mineralocorticoid receptor. *GR*: glucocorticoid receptor. *HSP*: heat shock protein. *GAPDH*: glyceraldehyde-3-phosphate dehydrogenase.

## Data Availability

The original contributions presented in the study are included in the article; further inquiries can be directed to the corresponding author.

## References

[1] Zhou X.Y., Wu Y.H., Zhou Y., Zhang Z., Chen G., Yu X.M., Tong J.G. (2024). Different dissolved oxygen effects on growth and O2-sensing pathway in juvenile bighead carp (*Hypophthalmichthys nobilis*). Aquaculture.

[2] Zhang K.S., Ye Z.Y., Qi M., Cai W.L., Saraiva J.L., Wen Y.C., Liu G., Zhu Z., Zhu S.M., Zhao J. (2025). Water Quality Impact on Fish Behavior: A Review From an Aquaculture Perspective. Rev. Aquac..

[3] Ott B.D., Chisolm D.O., Richardson B.M., Yamamoto F.Y., Goodman P., Reifers J.G., Bosworth B.G. (2026). Impact of diet, dissolved oxygen concentration, and stocking density on channel catfish (*Ictalurus punctatus*) production performance. J. World Aquac. Soc..

[4] He Y., Yu H.D., Zhang Z.Y., Zhang J.Y., Kang S.C., Zhang X.Z. (2022). Effects of chronic hypoxia on growth performance, antioxidant capacity and protein turnover of largemouth bass (*Micropterus salmoides*). Aquaculture.

[5] Liland N.S., Lai F.R.A., Sicuro A., Azevedo M.L., Araujo P., Hagen C., Sissener N.H., Soares F., Ronnestad I. (2026). On-growing Atlantic salmon (*Salmo salar* L.) acclimate behaviourally and physiologically to chronic hypoxia, but exhibit reduced feed intake, growth and lipid retention. Aquaculture.

[6] Williams B.L., Pintor L.M., Tiarks J., Gray S.M. (2024). Multiple stressors disrupt sex hormones and fitness outcomes: Effects of hypoxia and turbidity on an African cichlid fish. Conserv. Physiol..

[7] Sato E., Shimamura N., Saiki C., Sunada K., Miwa N., Xiao L. (2025). Hydrogen Gas Mitigates Acute Hypoxia-Induced Oxidative and Inflammatory Brain Injuries in Medaka (*Oryzias latipes*). Antioxidants.

[8] Zeng S.H., Feng L., Wu P., Liu Y., Ma Y.B., Ren H.M., Jin X.W., Zhou X.Q., Jiang W.D. (2025). Glutathione mitigates hypoxia-induced gill damage in juvenile grass carp (*Ctenopharyngodon idellus*) by alleviating endoplasmic reticulum stress and autophagy. J. Anim. Sci. Biotechnol..

[9] Li Y.X., Tan H.Q., Deng Y.X., Zhou D.Z., Zhu M. (2025). Hypoxia monitoring of fish in intensive aquaculture based on underwater multi-target tracking. Comput. Electron. Agric..

[10] Huang J., Older C.E., Heckman T.I., Jordan H.R., Griffin M.J., Allen P.J., Reifers J.G., Goodman P.M., Yamamoto F.Y. (2026). Autochthonous probiotic Lactococcus lactis MA5 improves recovery from acute hypoxia stress and resistance to Edwardsiella ictaluri in hybrid catfish (*Ictalurus punctatus* × *I. furcatus*). Fish Shellfish Immunol..

[11] Nogueira N., Ferreira M., Cordeiro N., Canada P. (2018). Quality parameters of wild white trevally (*Pseudocaranx dentex*) natural spawn kept in captivity. Aquaculture.

[12] Watanabe T., Vassallo-Agius R. (2003). Broodstock nutrition research on marine finfish in Japan. Aquaculture.

[13] Honryo T., Tsukada H., Kurata M., Katayama S., Agawa Y., Sawada Y. (2021). Suitable light intensity during the ontogenetic development of white trevally, Pseudocaranx dentex (Bloch and Schneider, 1801), larvae. Aquac. Res..

[14] Wang H., Li B.S., Yang L., Jiang C., Zhang T., Liu S.F., Zhuang Z.M. (2022). Expression profiles and transcript properties of fast-twitch and slow-twitch muscles in a deep-sea highly migratory fish, *Pseudocaranx dentex*. Peerj.

[15] Li B.S., Wang H., Yang L., Liu S.F., Zhuang Z.M. (2021). Complete Mitochondrial Genome of Pseudocaranx dentex (*Carangidae, Perciformes*) Provides Insight into Phylogenetic and Evolutionary Relationship among Carangidae Family. Genes.

[16] Wang H., Li B.S., Li J.F., Jiang C., Liu S.F., Zhuang Z.M. (2022). Label-Free Quantitative Proteomic Analysis Provides Insight Into the Differences Between Slow-Twitch Muscle and Fast-Twitch Muscle of Pseudocaranx dentex. Front. Mar. Sci..

[17] Wang H., Li A., An C.T., Che S., Huang R., Liu S.F., Zhuang Z.M. (2025). Transcriptome profiling and alternative splicing analysis of skeletal muscle in Pseudocaranx dentex: Insights into slow-twitch and fast-twitch muscle specialization. Int. J. Biol. Macromol..

[18] Wang H., Li B.S., Li A., An C.T., Liu S.F., Zhuang Z.M. (2024). Integrative Metabolomics, Enzymatic Activity, and Gene Expression Analysis Provide Insights into the Metabolic Profile Differences between the Slow-Twitch Muscle and Fast-Twitch Muscle of Pseudocaranx dentex. Int. J. Mol. Sci..

[19] Araki K., Nishiki I., Yoshida T. (2024). Characterization and Epidemiological Study of Newly Emerging Lactococcus garvieae Serotype III in Farmed Fish in Japan. Fish. Pathol..

[20] Oe R., Shirakashi S., Ishitani H., Carabott M.J., Chinh N.N. (2026). Morphological and molecular characterization of four Ceratomyxa species parasitizing marine fish in Japan including descriptions of three new species. Parasitol. Int..

[21] Li B.S., Wang H., Li A., An C.T., Zhu L., Liu S.F., Zhuang Z.M. (2022). The Landscape of DNA Methylation Generates Insight Into Epigenetic Regulation of Differences Between Slow-Twitch and Fast-Twitch Muscles in Pseudocaranx dentex. Front. Mar. Sci..

[22] Wang H., Li J.F., Li A., An C.T., Che S., Liu S.C., Liu S.F., Zhuang Z.M. (2025). Functional Roles and Regulatory Factors of Histidine-Related Compounds in Skeletal Muscle Metabolism of Teleosts with Distinct Swimming Strategies. Mar. Biotechnol..

[23] Jia Y., Wang J., Gao Y., Huang B. (2021). Hypoxia tolerance, hematological, and biochemical response in juvenile turbot (*Scophthalmus maximus*. L). Aquaculture.

[24] Wang F., Xie T., Wang J., Chen S., Liu Z., Jia Y. (2025). Hypoxia tolerance and physiological coping strategies in fat greenling (*Hexagrammos otakii*). Fish Physiol. Biochem..

[25] Verberk W., Sandker J.F., van de Pol I.L.E., Urbina M.A., Wilson R., McKenzie D.J., Leiva F.P. (2022). Body mass and cell size shape the tolerance of fishes to low oxygen in a temperature-dependent manner. Glob. Change Biol..

[26] McPhee D., Watson J.R., Harding D.J., Prior A., Fawcett J.H., Franklin C.E., Cramp R.L. (2023). Body size dictates physiological and behavioural responses to hypoxia and elevated water temperatures in Murray cod (*Maccullochella peelii*). Conserv. Physiol..

[27] Versteeg M.A., MacDonald C., Bennett-Smith M.F., Buston P.M., Rueger T. (2025). Individual clown anemonefish shrink to survive heat stress and social conflict. Sci. Adv..

[28] Richardson B.M., ul-Huda N., Ware C., Camus A.C., Older C.E., Yamamoto F.Y., Goodman P.M., Reifers J.G., Walker C.M., Stilwell J.M. (2025). Fingerling stocking size has no influence on proliferative gill disease severity in farm-raised Channel Catfish. J. Aquat. Anim. Health.

[29] von Herbing I.H. (2024). Energetic Costs of Stress in Developing Fishes: Quantifying Allostasis and Allostatic Load. Integr. Comp. Biol..

[30] Tsai M.H., Goh C.E., Lee M.H., Seah F., Lim M., Febriana E., Fu J.H., Yip J.K., Preshaw P., Toh S. (2025). Associations between perceived stress and glycemic measures: Gender and age as moderators. BMJ Open Diabetes Res. Care.

[31] Saraceni A., Rubin A.E., Wexler Y., Gothilf Y., Bovolin P., Zucker I. (2025). Simulated Environmental Nanoplastics Induce Zebrafish Developmental Toxicity and Stress Response. Acs Sustain. Chem. Eng..

[32] Pajand Z.O., Hosseinnia E., Pastaki N.J., Hallajian A., Yousefi A., Lebria A., Ashori A., Abdollahpour H. (2026). Size-dependent salinity tolerance in juvenile stellate sturgeon (*Acipenser stellatus*): Physiological and histopathological responses. Aquaculture.

[33] Wu C.B., Hou Z.G., Zhao X., Xu Y.H., Gong C.G. (2025). Single-cell RNA sequencing reveals heterogeneous response of interlamellar cell mass to hypoxic stress in crucian carp (*Carassius auratus*) gills. Fish Shellfish Immunol..

[34] Jia Y.D., Wang F.L., Gao Y.T., Qin H.Y., Guan C.T. (2023). Hypoxia stress induces hepatic antioxidant activity and apoptosis, but stimulates immune response and immune-related gene expression in black rockfish *Sebastes schlegelii*. Aquat. Toxicol..

[35] Tashakorizadeh M., Golkar P., Vahabi M.R., Ghorbanpour M. (2023). Physiological and biochemical mechanisms of grain yield loss in fumitory (*Fumaria parviflora* Lam.) exposed to copper and drought stress. Sci. Rep..

[36] Amaral D., Filipe D.M., Cavalheri T.F., Vieira L., Magalhães R.P., Belo I., Peres H., Ozório R.O.d.A. (2023). Solid-State Fermentation of Plant Feedstuff Mixture Affected the Physiological Responses of European Seabass (*Dicentrarchus labrax*) Reared at Different Temperatures and Subjected to Salinity Oscillation. Animals.

[37] Cao X.L., Cui H., Ji X.Y., Lu Y.Y.J., Kang Q.X., Lu R.H., Zhang Y.R., Xu X.X., Chen J.J. (2025). Branched-Chain Amino Acids Target miR-203a/fosb Axis to Promote Skeletal Muscle Growth in Common Carp (*Cyprinus carpio*). Aquac. Nutr..

[38] Deochand D.K., Dacic M., Bale M.J., Daman A.W., Chaudhary V., Josefowicz S.Z., Oliver D., Chinenov Y., Rogatsky I. (2024). Mechanisms of epigenomic and functional convergence between glucocorticoid- and IL4-driven macrophage programming. Nat. Commun..

[39] Liu Y., Wang J.Q., Ding J., Zhang Y.B., Hou C.C., Shen W.L., Wu X.F., Zhu J.Q. (2024). Effects of hypoxia stress on oxidative stress, apoptosis and microorganisms in the intestine of large yellow croaker (*Larimichthys crocea*). Aquaculture.

[40] Wu L.Q., Zhao P., Wu P., Jiang W.D., Liu Y., Ren H.M., Jin X.W., Zhou X.Q., Feng L. (2024). Curcumin attenuates ochratoxin A and hypoxia co-induced liver injury in grass carp (*Ctenopharyngodon idella*) by dual targeting endoplasmic reticulum stress and apoptosis via reducing ROS content. J. Anim. Sci. Biotechnol..

[41] Ali M.J., Tao Y.F., Li Y., Sayouh M.A., Lu S.Q., Qiang J., Xu P. (2024). Modulation of chronic hypoxia on ovarian structure, oxidative stress, and apoptosis in female Nile Tilapia (*Oreochromis niloticus*). Aquaculture.

